# Human cytomegalovirus protein UL42 antagonizes cGAS/MITA-mediated innate antiviral response

**DOI:** 10.1371/journal.ppat.1007691

**Published:** 2019-05-20

**Authors:** Yu-Zhi Fu, Yi Guo, Hong-Mei Zou, Shan Su, Su-Yun Wang, Qing Yang, Min-Hua Luo, Yan-Yi Wang

**Affiliations:** 1 Key Laboratory of Special Pathogens and Biosafety, Wuhan Institute of Virology, Chinese Academy of Sciences, Wuhan, China; 2 Medical Research Institute, School of Medicine, Wuhan University,Wuhan, China; Oregon Health and Sciences University, UNITED STATES

## Abstract

Cyclic GMP-AMP synthase (cGAS) senses viral DNA in the cytosol and then catalyzes synthesis of the second messenger cGAMP, which activates the ER-localized adaptor protein Mediator of IRF3 Activator (MITA) to initiate innate antiviral response. Human cytomegalovirus (HCMV) proteins can antagonize host immune responses to promote latent infection. Here, we identified HCMV UL42 as a negative regulator of cGAS/MITA-dependent antiviral response. UL42-deficiency enhances HCMV-induced production of type I interferons (IFNs) and downstream antiviral genes. Consistently, wild-type HCMV replicates more efficiently than UL42-deficient HCMV. UL42 interacts with both cGAS and MITA. UL42 inhibits DNA binding, oligomerization and enzymatic activity of cGAS. UL42 also impairs translocation of MITA from the ER to perinuclear punctate structures, which is required for MITA activation, by facilitating p62/LC3B-mediated degradation of translocon-associated protein β (TRAPβ). These results suggest that UL42 can antagonize innate immune response to HCMV by targeting the core components of viral DNA-triggered signaling pathways.

## Author summary

Recognition of viral DNA by the cytosolic DNA sensor cGAS and subsequent induction of type I IFNs via the cGAS-MITA signaling axis are important for host antiviral innate immunity. The human cytomegalovirus (HCMV) causes complications in immunodeficient populations and is a major cause of birth defects. It is known that HCMV suppresses innate immunity, which is pivotal for establishing immune evasion and latent infection. In this study, we found that HCMV protein UL42 inhibits innate antiviral responses thus promotes HCMV replication. UL42 functions by targeting cGAS and MITA through distinct mechanisms. UL42 inhibits cGAS activation by interrupting its DNA binding and oligomerization, while it targets MITA by interfering trafficking of MITA from the ER to perinuclear punctate structures, a process required for MITA activation. These findings defined an important mechanism for HCMV immune evasion, which may provide a therapeutic target for the treatment of HCMV infection.

## Introduction

The innate immune system is the first line of host defense against microbial infection. Upon microbial infection, cellular pattern recognition receptors (PRRs) recognize structurally conserved microbial components called pathogen-associated molecular patterns (PAMPs), which triggers a series of signaling events that lead to the induction of type I interferons (IFNs), pro-inflammatory cytokines and other downstream effectors. These effectors mediate the inhibition of microbial replication, clearance of infected cells and facilitation of adaptive immune response to eliminate infected pathogens [1–4].

Among the PRRs, cyclic guanosine monophosphate-adenosine monophosphate (cGAMP) synthase (cGAS) has been demonstrated as a general cytosolic DNA sensor in response to DNA virus infection in various cell lines and in mice [5, 6]. cGAS recognizes double-stranded DNA (dsDNA) and utilizes ATP and GTP to synthesize the second messenger cGAMP [7]. cGAMP binds to the ER-localized adaptor protein MITA [8], which is also designated as STING, ERIS, and MPYS [9–12]. The cGAMP-bound MITA traffics from the ER to the Golgi apparatus via the inactive rhomboid protein 2 (iRhom2) and TRAPβ containing translocon complex [13–16], and then further to the Sec5-containing perinuclear punctate structures [10]. During the trafficking processes, MITA recruits TANK-binding kinase 1(TBK1) and interferon regulatory factor 3 (IRF3), leading to induction of type I interferons and other antiviral effectors [17].

In addition to trafficking, the functions of MITA are also regulated by several post-translational mechanisms. The E3 ubiquitin ligases TRIM32 and TRIM56 can catalyze K63-linked polyubiquitination of MITA and promote the recruitment of TBK1 to MITA, thereby positively regulating innate immune responses [18, 19]. In addition, the ER-associated E3 ligase AMFR mediates K27-linked polyubiquitination of MITA, providing a scaffold to recruit TBK1 and IRF3 [20, 21].

The human cytomegalovirus (HCMV), a member of the beta herpesvirus family, is a typical dsDNA virus that encodes over 200 proteins [22]. HCMV causes global epidemics and complications in AIDS patients and organ transplant recipients and is a major cause of birth defects [23]. HCMV infection is also associated with inflammatory and proliferative diseases such as certain cardiovascular diseases and cancers [24]. However, there is no vaccine to prevent HCMV infection, and the drugs currently approved for the treatment of HCMV infectious diseases suffer from low bioavailability, toxicity, and the generation of resistant viruses [25]. HCMV proteins could suppress cellular and organismal defenses, which are pivotal for establishing immune evasion and latent infection [26]. Therefore, HCMV has become an ideal model for the study of viral immune evasion due to its multiple strategies to modulate host innate and adaptive responses [27]

Similar to many other DNA viruses, the cGAS-MITA axis also plays a crucial role in HCMV-induced host antiviral defense [28, 29]. Meanwhile, HCMV have evolved various mechanisms to antagonize this signaling pathway for efficient infection and replication [30, 31]. For example, it has been demonstrated that HCMV tegument protein UL82 contributes to HCMV immune evasion by inhibiting the cellular trafficking and activation of MITA to evade antiviral immunity [15]. UL31 inhibits DNA sensing of cGAS to mediate immune evasion [32]. UL83 inhibits gamma-interferon-inducible protein 16 (IFI16)- and cGAS-mediated DNA sensing for immune evasion [33, 34]. Whether other HCMV proteins are involved in antagonization of innate antiviral response are unclear.

In this study, we identified HCMV UL42 as an inhibitor of innate antiviral response. UL42 is classified as a CMV-specific but function-unknown gene, which consists of 124 amino acids and partially localized at the trans-Golgi network and cytoplasmic vesicles [35, 36]. In addition, UL42 has a C-terminal hydrophobic domain predicted to be transmembrane domain and two PPXY motifs in its N terminus. Our results suggest that UL42 inhibits cGAS activation and impairs the trafficking of MITA, thereby contributes to HCMV evasion of innate antiviral responses.

## Results

### HCMV UL42 suppresses viral DNA-triggered signaling

**Fig 1:**
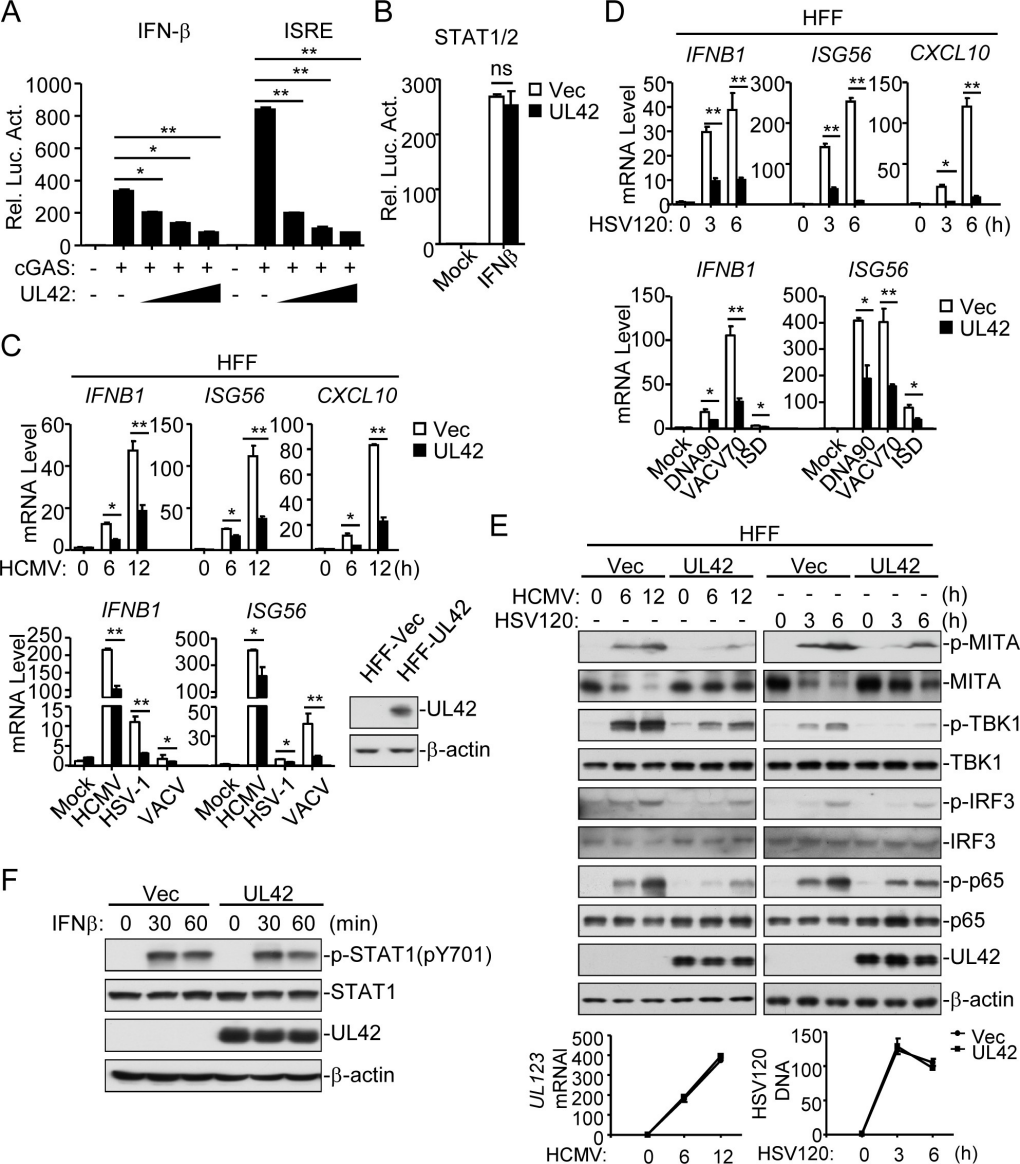
Identification of HCMV UL42 as an inhibitor of DNA-triggered signaling. (A) HCMV UL42 inhibits cGAS-MITA-induced IFNβ promoter and ISRE activation in a dose-dependent manner. HEK293T/MITA cells (1x10^5^) were transfected with the IFNβ promoter (0.05 μg) or ISRE (0.03 μg) reporter plasmid, and expression plasmids for cGAS (0,01 μg) and increased amounts of UL42 (0, 0.025, 0.05, and 0.1 μg) for 20 hrs before luciferase assays. (B) Effects of UL42 on IFN-β-induced STAT1/2 activation. HEK293 cells (1x10^5^) were transfected with STAT1/2 reporter (0.005 μg) and UL42 expression (0.05 μg) plasmids for 20 hrs. The cells were then untreated or treated with IFN-β for 12 hrs before luciferase assays. (C) HCMV UL42 inhibits HCMV-, HSV-1-, and VACV-induced transcription of antiviral genes in HFFs. UL42-stable HFFs (4x10^5^) were un-infected or infected with HCMV (MOI = 1), HSV-1 (MOI = 1), or VACV (MOI = 1) for the indicated times (upper histographs) or 12 h (lower histographs) before qPCR analysis. The immunoblots show the expression levels of UL42 in the HFF-UL42 stable cell lines. (D) HCMV UL42 inhibits dsDNA-induced transcription of antiviral genes in HFFs. UL42 stable HFFs (4x10^5^) were transfected with HSV120 (2 μg), DNA90 (2 μg), VACV70 (2 μg), or ISD (2 μg) for the indicated times before qPCR analysis. (E) UL42 impairs HCMV- and HSV120-induced phosphorylation of downstream components. UL42 stable HFFs (4x10^5^) were infected with HCMV (MOI = 1) or transfected with HSV120 (2 μg/ml) for the indicated times before immunoblot analysis. The lower panels are results of qPCR analysis for HCMV UL123 mRNA or HSV120 DNA. (F) Effect of UL42 on IFN-β-induced phosphorylation of STAT1. UL42 stable HFFs (4x10^5^) were untreated or treated with IFN-β (100 ng/ml) for the indicated times before immunoblot analysis. Graphs show mean ± SD, n = 3. *p<0.05, **p<0.01 (unpaired t test).

Previously, we performed systematic screens for HCMV proteins that can inhibit DNA-triggered activation of interferon-stimulated response element (ISRE, which is bound by activated IRF3) by reporter assays and identified UL42 as a candidate protein [15]. Reporter assays indicated that overexpression of UL42 inhibited cGAS-induced activation of the IFN-β promoter (which is driven by ISRE and κB enhancers) and ISRE in a dose-dependent manner in HEK293T cells stably expressing MITA (HEK293T/MITA) (Fig 1A), but did not affect IFN-β-induced activation of signal transducer and activator of transcription 1/2 (STAT1/2) (Fig 1B). Previously, it has been shown that the human primary foreskin fibroblasts (HFFs) can express downstream antiviral genes in response to HCMV infection [33, 37]. Our results indicated that HCMV AD169 strain could infect HFF cells, but could not infect endothelial HUVEC, epithelial HEK293T and Ea. hy926 cells (S1A & S1B Fig). We established HFF cell lines that stably express UL42 (HFF-UL42) by lentiviral-mediated transduction (Fig 1C). qPCR analysis indicated that induction of antiviral genes including *IFNB1*, *ISG56* and *CXCL10* following infection with the DNA viruses HCMV, herpes simplex virus 1 (HSV-1), and vaccinia virus (VACV) was inhibited in HFF-UL42 as compared to empty vector-transduced control cells (HFF-Vec) (Fig 1C). In addition, transcription of genes induced upon transfection of dsDNAs, including 120-mer dsDNA representing the genome of HSV-1 (HSV120), dsDNA of approximately 90 bp (dsDNA90), 70-mer dsDNA representing the genome of vaccinia virus (VACV70) and 45-mer interferon stimulatory DNA (ISD45), was impaired in HFF-UL42 cells (Fig 1D). Since phosphorylation of MITA, TBK1, IRF3, and p65 are hallmarks of cGAS/MITA-mediated signaling, we further examined the effects of UL42 on these events. Consistently, ectopic expression of UL42 dramatically inhibited phosphorylation of MITA, TBK1, IRF3 and RelA (p65) in response to HCMV and HSV120 (Fig 1E). In contrast, UL42 did not have marked effects on phosphorylation of STAT1 induced by IFN-β in HFFs (Fig 1F). In these experiments, MITA was down-regulated after HCMV infection and HSV120 stimulation, which is a mechanism of timely termination of innate antiviral response to avoid immune damage [13, 38, 39]. As previously reported, down-regulation of MITA is dependent on its activation and happens during its trafficking from the ER to the perinuclear punctate structures [13, 30, 40, 41]. Notably, the down-regulation of MITA following HCMV infection was inhibited in HFF-UL42, suggesting a role of UL42 in MITA-mediated signaling.

### UL42-deficiency potentiates HCMV-triggered antiviral response

**Fig 2:**
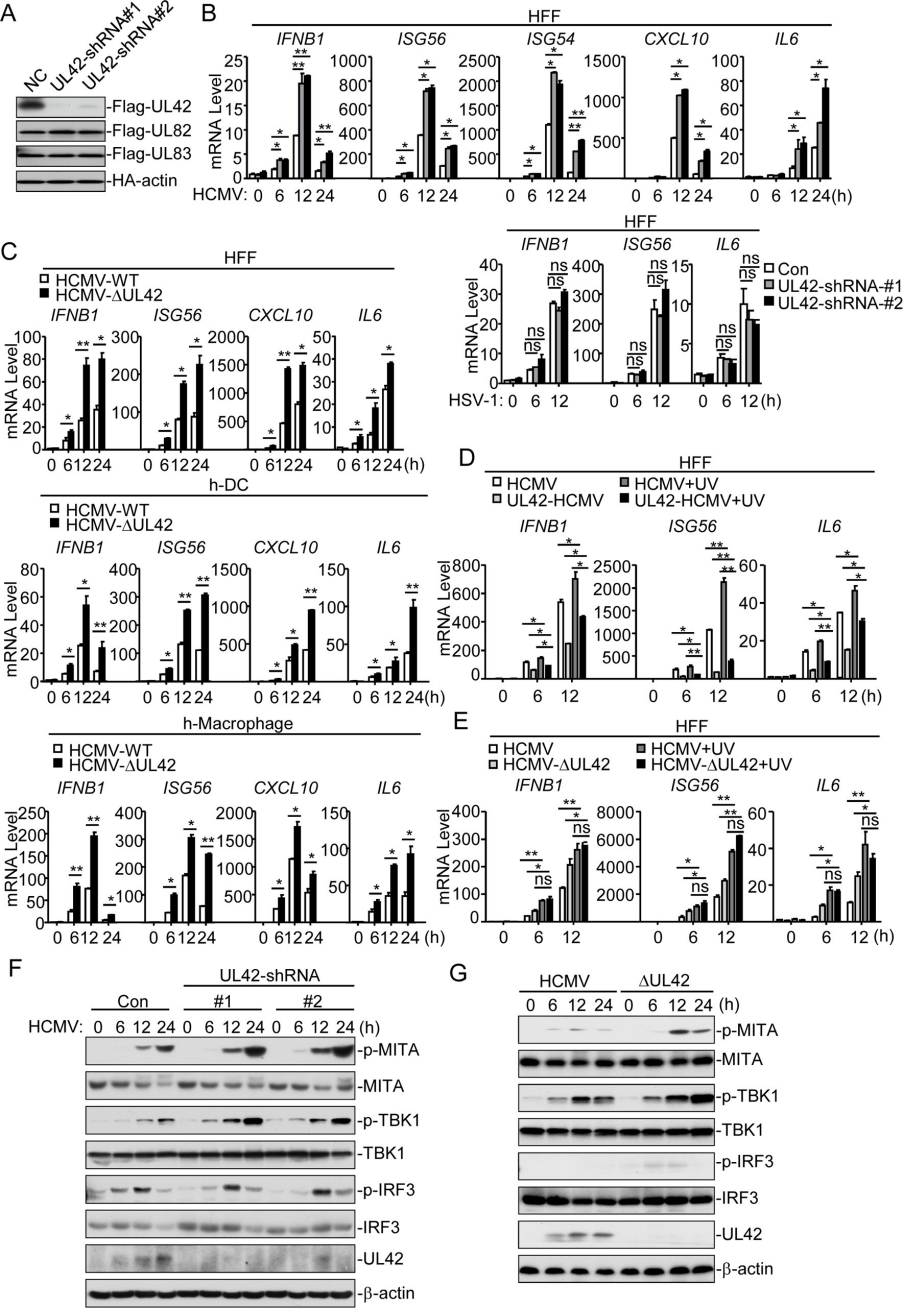
UL42-deficiency potentiates innate antiviral response to HCMV. **(A)** UL42-shRNAs inhibited expression of UL42. HEK293 cells (2×10^5^) were transfected with Flag-UL42, Flag UL82, Flag-UL83 or HA-actin expression plasmids and the indicated shRNA plasmids (2 μg each) for 20 hrs before immunoblot analysis. (B) Effects of UL42-shRNAs on HCMV- and HSV-1-induced transcription of downstream antiviral genes. UL42-shRNA stable HFFs (4x10^5^) were infected with HCMV (MOI = 1) or HSV-1 (MOI = 1) for the indicated times before qPCR analysis. (C) HCMV-ΔUL42 virus elicits stronger innate immune response than wild-type HCMV. The indicated cells (4x10^5^) were infected with wild-type HCMV (MOI = 1) or HCMV-ΔUL42 (MOI = 1) for the indicated times before qPCR analysis. (D) Inhibition of UL42 on *IFNB1*, *ISG56*, *and IL6 t*ranscription induced by UV-inactivated HCMV. Control or UL42 stable cells (4x10^5^) were infected with wild-type or UV-inactivated HCMV before qPCR analysis. (E) Effects of UL42-deficiency on transcription induced by UV-inactivated wild-type or UL42-deficient HCMV. The indicated cells (4x10^5^) were infected with untreated or UV-inactivated wild-type or UL42-deficient HCMV (MOI = 1) for the indicated times before qPCR analysis. (F) Effects of UL42-shRNAs on HCMV-induced phosphorylation of downstream components. UL42-shRNA stable HFFs (4x10^5^) were infected with HCMV (MOI = 1) for the indicated times before immunoblot analysis. (G) Effects of UL42-deficiency on phosphorylation of downstream components. The indicated cells (4x10^5^) were infected with wild-type HCMV (MOI = 1) or HCMV-ΔUL42 (MOI = 1) for the indicated times before immunoblot analysis. Graphs show mean ± SD, n = 3. *p<0.05, **p<0.01(unpaired t test).

To investigate the roles of endogenous UL42 in innate antiviral response to HCMV, we constructed two UL42-shRNA plasmids that could specifically knock down the expression of UL42, but not other HCMV genes (Fig 2A). qPCR analysis indicated that knockdown of UL42 promoted HCMV- but not HSV-1-induced transcription of *IFNB1*, *ISG56*, *ISG54*, *CXCL10*, and *IL6* genes at 6, 12, and 24 hr post-infection in HFFs (Fig 2B). These results suggest that UL42-deficiency promotes innate antiviral response.

To further confirm the role of UL42, we generated UL42-deficient HCMV (HCMVΔUL42) by CRISPR/Cas9 technology. We next examined the expression of downstream antiviral genes in cells infected with wild-type HCMV or HCMV-ΔUL42. Consistently, mRNA levels of *IFNB1*, *ISG56*, C*XCL10*, and *IL6* genes induced by HCMV-ΔUL42 were significantly higher than those induced by HCMV-WT at 6, 12, and 24 hr post-infection in HFFs, human primary monocyte-derived dendritic cells and macrophages (Fig 2C). In addition, UV-inactivated HCMV, which does not undergo viral transcription and translation after infection, induced higher levels of *IFNB1*, *ISG56*, and *IL6* mRNA than un-treated HCMV. In these experiments, UL42 also inhibited transcription of *IFNB1*, *ISG56*, and *IL6* induced by UV-inactivated HCMV (Fig 2D). We also examined the mRNA levels of UV-treated or untreated HCMV. qPCR assays indicated that HCMV were inactivated by UV treatment (S2 Fig). Furthermore, HFFs infected with UV-inactivated HCMV-WT or HCMV-ΔUL42 showed little difference on mRNA level of *IFNB1*, *ISG56*, and *IL6* (Fig 2E), suggesting that UL42 directly affects HCMV-induced transcription of downstream antiviral genes. Consistently, knockdown of UL42 increased HCMV-induced phosphorylation of MITA, TBK1, and IRF3 in HFFs (Fig 2F). In addition, phosphorylation of MITA, TBK1, and IRF3 was increased following infection with HCMV-ΔUL42 compared to wild-type HCMV (Fig 2G). Taken together, these results suggest that UL42 plays a critical role in the inhibition of HCMV DNA-triggered induction of downstream antiviral genes.

### UL42 mediates HCMV evasion of innate immune response

**Fig 3:**
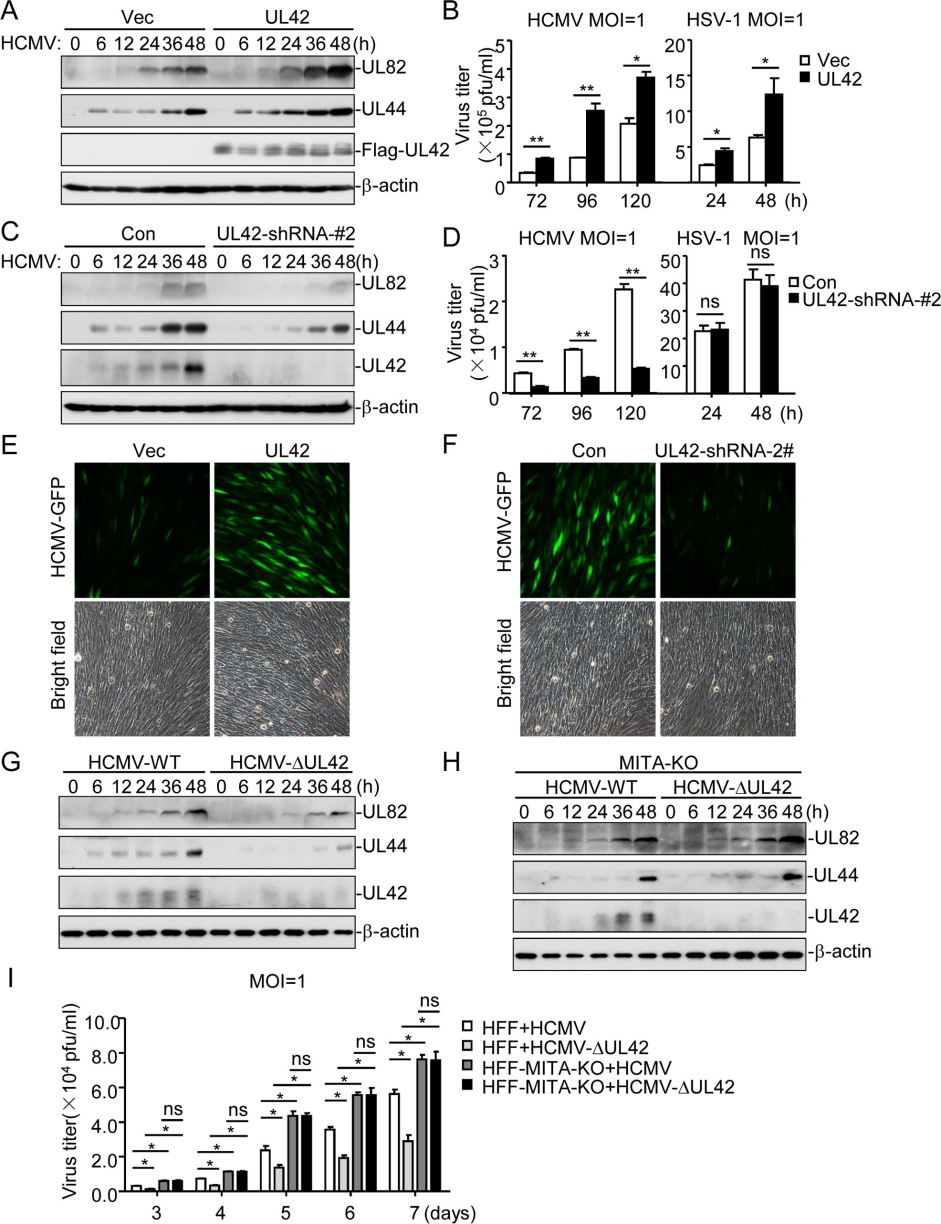
Roles of UL42 on HCMV replication. (A) UL42 enhances HCMV replication in early phase of infection. The indicated cells (4x10^5^) were infected with HCMV (MOI = 1) for the indicated times before immunoblotting analysis. (B) UL42 enhances HCMV and HSV-1 replication in late phase of infection. The indicated cells (1x10^6^) were infected with HCMV (MOI = 1) or HSV-1 (MOI = 1), and the supernatants were harvested at the indicated times post infection for measurements of viral titers with standard TCID50 or plaque assays. (C) Effects of UL42-knockdown on HCMV replication in early phase of infection. The indicated cells (4x10^5^) were infected with HCMV (MOI = 1) for the indicated times before immunoblotting analysis. (D) Effects of UL42-knockdown on HCMV and HSV-1 replication in late phase of infection. The indicated cells (1x10^6^) were infected with HCMV (MOI = 1) or HSV-1 (MOI = 1), and the supernatants were harvested at the indicated times post infection for measurements of viral titers with standard TCID50 or plaque assays. (E) Effects of UL42 on HCMV-GFP replication. HFFs transduced with UL42 or a control vector were left uninfected or infected with HCMV-GFP (MOI = 0.1) for 3 days before fluorescent microscopy. (F) Effects of UL42-knockdown on HCMV replication. The indicated cells (5x10^4^) were infected with HCMV-GFP (MOI = 1) for 3 days before fluorescent microscopy. (G) Effects of UL42-deficiency on HCMV replication in HFFs. Cells (4x10^5^) were infected with HCMV (MOI = 1) or HCMV-ΔUL42 (MOI = 1) for the indicated times before immunoblotting analysis. (H) Effects of UL42-deficiency on HCMV replication in HFF-MITA-KO cells. Cells (4x10^5^) were infected with HCMV (MOI = 1) or HCMV-ΔUL42 (MOI = 1) for the indicated times before immunoblotting analysis. (I) Effects of UL42-deficiency on HCMV replication. The indicated cells (1x10^6^) were infected with wild-type HCMV or HCMV-ΔUL42 for the indicated days. The supernatants were then harvested for measurements of the viral titers with standard TCID50 assays. Graphs show mean ± SD, n = 3. *p<0.05, **p<0.01(unpaired t test).

Since UL42 antagonizes innate antiviral response, we further investigated its functions in HCMV immune evasion. Overexpression of UL42 markedly enhanced the replication of HCMV and HSV-1 (Fig 3A & 3B), whereas knockdown of UL42 inhibited replication of HCMV but not HSV-1 (Fig 3C & 3D). Fluorescence microscopy experiments indicated that overexpression of UL42 markedly enhanced replication of GFP-tagged HCMV (HCMV-GFP) [42] (Fig 3E), whereas knockdown of UL42 inhibited replication of HCMV-GFP in HFF cells (Fig 3F). These results suggest that UL42 contributes to HCMV immune evasion.

It has been shown that MITA/STING is a pivotal adaptor protein for viral DNA-induced expression of downstream antiviral genes [3, 8, 10, 43], which is also essential for innate immune response to HCMV [33]. Our results also indicated that MITA-deficiency inhibited HCMV-induced transcription of downstream antiviral genes (S3A Fig). However, knockdown of RIG-I or TLR9 did not affect HCMV-induced transcription of *IFNB1*genes in HFF cells (S3B & S3C Fig). We found that replication of HCMV-ΔUL42 was decreased in comparison with wild-type HCMV in HFF-WT cells (Fig 3G), but replications of both HCMV-ΔUL42 and wild-type HCMV were identical at early phase (6–48 hr) of infection in MITA-deficient cells (Fig 3H). Consistently, the progeny virions of HCMV-ΔUL42 were lower than wild-type HCMV in control cells, but they were identical in MITA-knockout cells at late phase (3–7 d) of infection (Fig 3I). Interestingly, both wild-type HCMV and HCMV-ΔUL42 production in MITA-knockout cells was increased in comparison to wild-type cells, consistent with a critical role of MITA in innate antiviral response (Fig 3H). These results suggest that UL42 plays a direct role in evasion of innate antiviral response and contribute to the replication of HCMV.

### UL42 acts at the levels of cGAS and MITA

**Fig 4:**
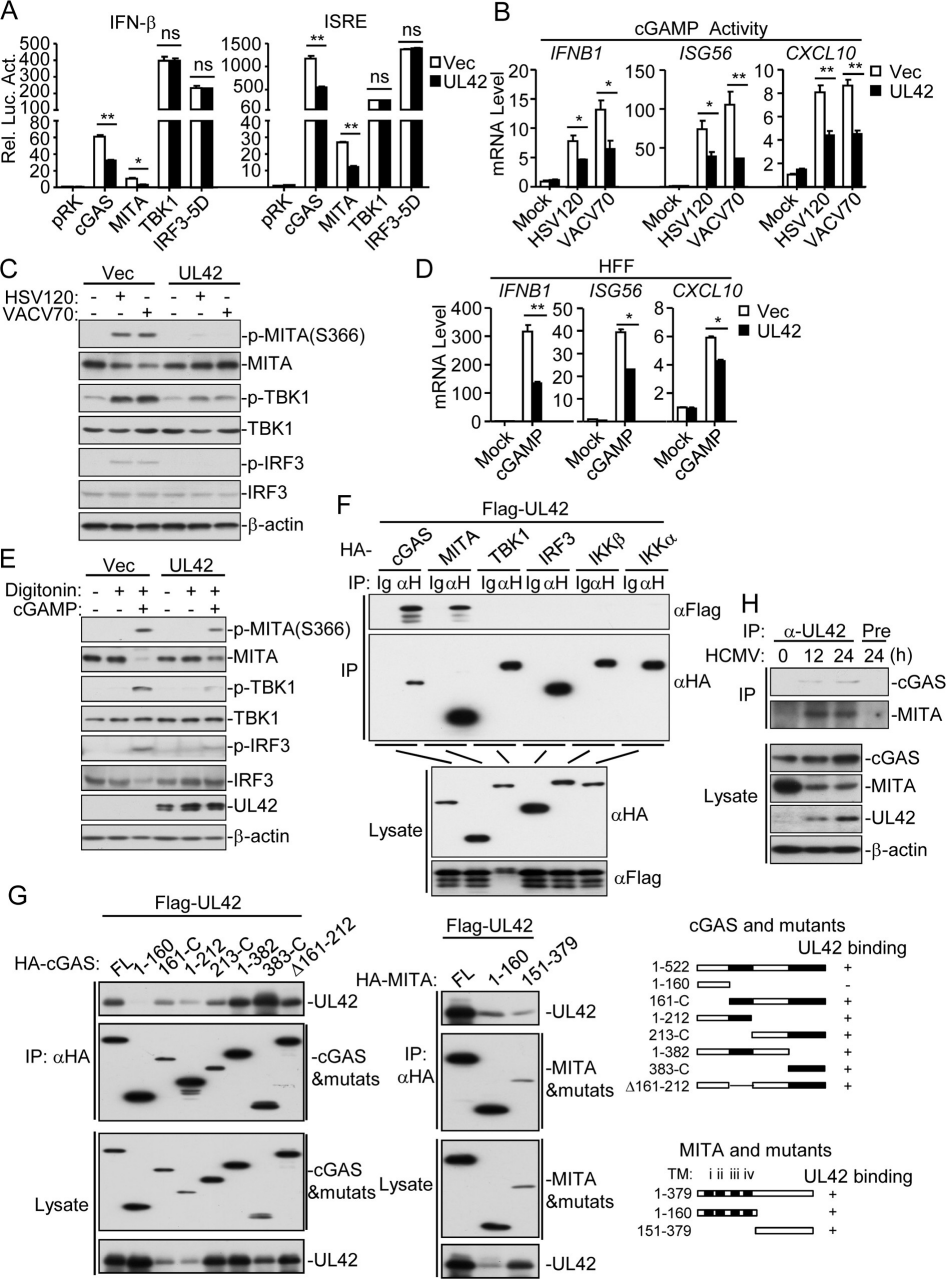
UL42 associates with cGAS and MITA. (A) Effects of UL42 on IFN-β and ISRE activation mediated by various components. HEK293T/MITA cells (1x10^5^) were transfected with the IFN-β promoter (0.05 μg) or ISRE reporter (0.05 μg), UL42 and the indicated expression plasmids (0.05 μg each) for 20 hrs before luciferase assays. (B) Effects of UL42 on cGAMP synthesis induced by transfected HSV120 or VACV70. HFF-Vec and HFF-UL42 cells (1x10^7^) were transfected with HSV120 (3 μg) or VACV70 (3 μg) for 4 hr, and then cell extracts containing cGAMP were delivered to digitonin-permeabilized HFFs for 4 hr before qPCR analysis. (C) Effects of UL42 on cGAMP synthesis induced by transfected HSV120 or VACV70. HFF-Vec and HFF-UL42 cells (1x10^7^) were mock-transfected or transfected with HSV120 (10 μg) or VACV70 (10 μg) for 4 hr, and then cell extracts containing cGAMP were delivered to digitonin-permeabilized HFFs for 4 hr before immunoblotting analysis. (D) UL42 inhibits cGAMP-induced transcription of antiviral genes in HFFs. Control or UL42-tranduced HFFs (4x10^5^) were transfected with cGAMP (0.2 μg) for 4 hr before qPCR analysis. (E) UL42 inhibits cGAMP-induced transcription of antiviral genes in HFFs. Control or UL42-tranduced HFFs (4x10^5^) were transfected with cGAMP (0.2 μg) for 4 hr before immunoblotting analysis. (F-G) Association of UL42 with cGAS and MITA. HEK293T cells (2x10^6^) were transfected with the indicated plasmids (5 μg each) for 20 hr before coimmunoprecipitation and immunoblotting analysis with the indicated antibodies. (H) Association of endogenous UL42 with cGAS and MITA in HFFs. The HFF cells (3x10^7^) were left untreated or infected with HCMV for 12 or 24 hr before coimmunoprecipitation and immunoblotting analysis with the indicated antibodies. Graphs show mean ± SD, n = 3. *p<0.05, **p<0.01 (unpaired t test).

Next, we investigated the molecular mechanisms on the negative regulatory role of UL42 in innate antiviral response. Reporter assays indicated that UL42 inhibited cGAS- and MITA- but not TBK1- or IRF3-5D (an active mutant of IRF3)-mediated activation of the IFN-β promoter and ISRE (Fig 4A). As previously described [7, 15, 32, 38], the extracts from DNA-transfected cells contain cGAMP, which trigger induction of *IFNB1*, *ISG56*, and *CXCL10* genes. To elucidate the mechanisms on how UL42 antagonizes innate antiviral response, we firstly examined whether UL42 affects cGAMP synthesis. Overexpression of UL42 impaired both HSV120- and VACV70-induced production of cGAMP (Fig 4B) and phosphorylation of MITA, TBK1 and IRF3 in HFFs (Fig 4C). These results suggest that UL42 is important for inhibiting viral DNA-induced cGAS activation. Interestingly, UL42 also dramatically inhibited cGAMP-induced transcription of downstream antiviral genes such as *IFNB1*, *ISG56*, and *CXCL10* (Fig 4D). cGAMP-induced phosphorylation of MITA, TBK1, and IRF3 in HFF-UL42 cells were decreased in comparison to HFF-Vec cells (Fig 4E). These results suggest that UL42 targets at steps both upstream and downstream of cGAMP in the cGAS-cGAMP-MITA signal pathway.

We next determined whether UL42 is associated with signaling components in dsDNA-triggered pathways. Co-immunoprecipitation experiments indicated that UL42 was associated with both cGAS and MITA, but not TBK1, IRF3, IKKβ or IKKα in overexpression system (Fig 4F). Domain mapping experiments indicated that the C-terminal fragment (aa161-522) of cGAS interacted with UL42, and both N-terminal fragment (aa1-160) and C-terminal fragment (161–379) of MITA could independently interact with UL42 (Fig 4G). Endogenous co-immunoprecipitation experiments indicated that UL42 was associated with both cGAS and MITA following HCMV infection (Fig 4H). These results suggest that UL42 targets both cGAS and MITA for antagonizing innate antiviral response.

### UL42 impairs DNA binding and oligomerization of cGAS

**Fig 5:**
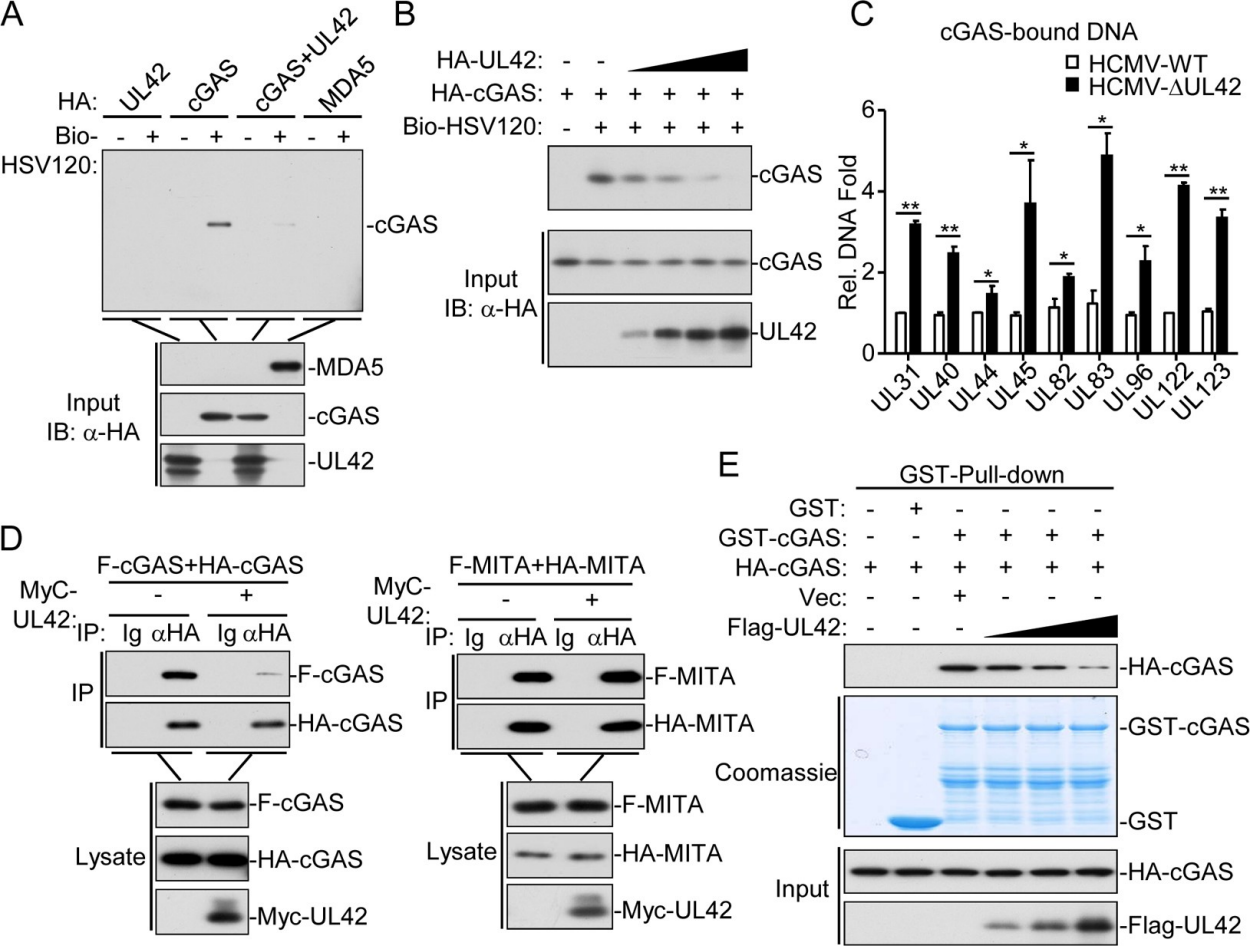
UL42 inhibits DNA binding and oligomerization of cGAS. (A&B) UL42 impairs the binding of cGAS to dsDNA. HEK293T cells (2×10^6^) were transfected with the indicated plasmids (2 μg each, except for UL42 in (B), where increased amounts of 1, 2, 3, and 4 μg were used as indicated). Twenty hours later, the cell lysates were incubated with the indicated biotinylated nucleic acids and streptavidin-Sepharose beads for in vitro pull-down assays. The bound proteins were then analyzed by immunoblots with anti-HA. (C) Quantification of cGAS-bound viral DNAs in cells infected with wild-type or UL42-deficient HCMV. HFFs (2x10^6^) were infected with HCMV-WT or HCMV-ΔUL42 (MOI = 1) for 12 hr. The cell lysates were immunoprecipitated with anti-cGAS and cGAS-bound DNAs were extracted and analyzed by qPCR analyses with primers for the indicated viral genes. (D) Effects of UL42 on self-association of cGAS and MITA. HEK293 cells (2×10^6^) were transfected with the indicated plasmids for 20 hr before co-immunoprecipitation and immunoblotting analysis with the indicated antibodies. (E) Effects of UL42 on self-association of cGAS. HEK293T cells (2×10^6^) were transfected with the indicated plasmids (2 μg each, except for UL42 was transfected with increased amounts of 1, 2, 3, and 4 μg) for 20 hrs before pull-down assays analysis with the indicated antibodies. Graphs show mean ± SD, n = 3. *p<0.05, **p<0.01 (unpaired t test).

Since UL42 interacts with cGAS, we next determined whether UL42 affects cGAS binding to DNA. As shown in Fig 5A, both UL42 and the RNA sensor MDA5 did not bind to HSV120 DNA in pull-down assays. However, UL42 dramatically inhibited the binding of cGAS to HSV120 DNA (Fig 5A). The inhibitory effect of UL42 on cGAS binding to DNA was dose-dependent (Fig 5B). In addition, levels of viral DNA bound by endogenous cGAS were higher in HFFs infected with HCMV-ΔUL42 in comparison with wild-type HCMV (Fig 5C). These results suggest that UL42 impairs cGAS binding to DNA. Previously, it has been shown that cGAS self-association and oligomerization are important for its activation after binding to dsDNA [44, 45]. Co-immunoprecipitation experiments indicated that UL42 inhibited self-association of cGAS but not MITA (Fig 5D). Consistently, UL42 markedly inhibited self-association of cGAS in a dose-dependent manner in pull-down assays (Fig 5E). However, overexpression of UL42 did not affect polyubiquitination of cGAS (S4A Fig). Collectively, these results suggest that UL42 impairs synthesis of cGAMP by inhibiting DNA binding and oligomerization of cGAS.

### UL42 impairs trafficking of MITA and promotes degradation of TRAPβ

**Fig 6:**
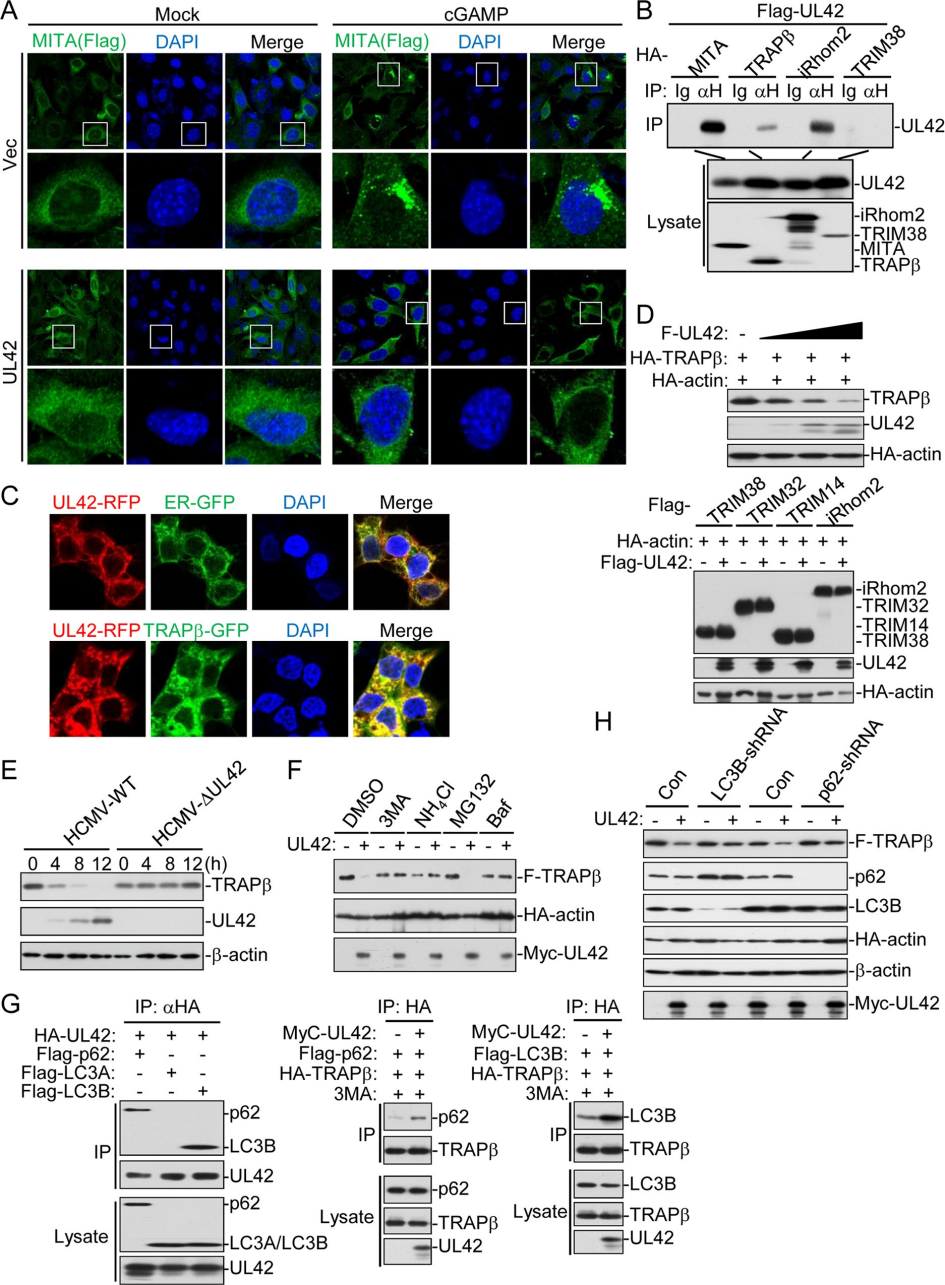
UL42 impairs the trafficking of MITA by promoting p62-LC3B- mediated autophagy degradation of TRAPβ. (A) UL42 impairs cGAMP-triggered trafficking of MITA. UL42 stably-transduced *MITA*^-/—^MLF-MITA-Flag cells (2x10^5^) were transfected with cGAMP (0.1 μg) for 4 hr before confocal microscopy. (B) Association of UL42 with MITA, TRAPβ, and iRhom2. HEK293T cells (2x10^6^) were transfected with the indicated plasmids (5 μg each) for 20 hr before coimmunoprecipitation and immunoblotting analysis with the indicated antibodies. (C) UL42 is co-localized with the ER and TRAPβ. HEK293 (5x10^4^) were transfected with UL42-RFP, TRAPβ**-**GFP or ER-GFP (1 μg each) as indicated for 18 hr before confocal microscopy. (D) UL42 promotes degradation of TRAPβ but not TRIM38, TRIM32, TRIM14, and iRhom2. HEK293T cells (2x10^5^) were transfected with the indicated plasmids (2 μg each) for 20 hr before immunoblotting analysis with the indicated antibodies. (E) HCMV-WT but not HCMV-ΔUL42 induced degradation of TRAPβ. The HFF-TRAPβ cells (4x10^5^) were infected with wild-type or UL42-deficient HCMV (MOI = 1) for the indicated times before immunoblotting analysis with the indicated antibodies. (F) Effects of different inhibitors on UL42-mediated destabilization of TRAPβ. HEK293 cells (5x10^5^) were transfected with the indicated plasmids (2 μg each) for 12 hours and then treated with the indicated inhibitors for 6 hours before immunoblotting analysis with the indicated antibodies. (G) UL42 increases the association of TRAPβ with LC3B and p62. HEK293T cells (5x10^6^) were transfected with the indicated plasmids (5 μg each) for 20 hrs before coimmunoprecipitation and immunoblot analysis with the indicated antibodies.(H) Knockdown of p62 or LC3B inhibits UL42-mediated destabilization of TRAPβ. The HEK293T-control, HEK293T-LC3B-shRNA, or HEK293T-p62-shRNA cells (5x10^5^) were transfected with the indicated plasmids (2 μg each) for 12 hours and then treated with the indicated inhibitors for 6 hours before immunoblotting analysis with the indicated antibodies.

Previously, it has been shown that self-association and oligomerization of MITA are crucial for MITA-mediated signaling [46]. As shown above, UL42 did not affect self-association (Fig 5D) or polyubiquitination (S4B Fig) of MITA. Interestingly, confocal microscopy indicated that UL42 inhibited accumulation of MITA in perinuclear punctate structures induced by cGAMP (Fig 6A), which is a key marker for MITA activation.

Previous studies have demonstrated that iRhom2 and TRAPβ containing complex is critically involved in MITA trafficking after viral infection [13, 14]. We found that UL42 was associated with TRAPβ and iRhom2 but not TRIM38, which mediates MITA sumoylation [38] (Fig 6B). Confocal microscopy confirmed that UL42 was colocalized with TRAPβ and the ER (Fig 6C). Interestingly, we found that UL42 promoted degradation of TRAPβ in a dose-dependent manner in overexpression experiments, but did not affect the stability of iRhom2, TRIM38, TRIM32 or TRIM14 (Fig 6D). Consistently, UL42-deficiency inhibited HCMV-induced degradation of TRAPβ in HFFs (Fig 6E). Taken together, these results suggest that UL42 impairs the trafficking of MITA after viral infection by promoting degradation of the translocon complex protein TRAPβ.

Two major systems exist for protein degradation, including the ubiquitin-proteasome and autophagy-lysosome pathways. To investigate the mechanisms responsible for UL42-mediated degradation of TRAPβ, we treated HEK293T-UL42 and HEK293T-Vec cells with various inhibitors for protein degradation pathways. The results indicated that UL42-mediated degradation of TRAPβ could be inhibited by the lysosomal inhibitor NH_4_Cl and the autophagic inhibitors 3MA and bafilomycin, but not the proteasomal inhibitor MG132 (Fig 6F), suggesting that UL42 mediates degradation of TRAPβ via an autophagic lysosomal pathway. In co-immunoprecipitation experiments, UL42 interacted with p62 and LC3B, two essential components involved in autophagic lysosomal degradation, and increased the associations of p62-TRAPβ or LC3B-TRAPβ (Fig 6G). Consistently, knockdown of p62 or LC3B inhibited UL42-mediated degradation of TRAPβ (Fig 6H). These results suggest that UL42 impairs the trafficking of MITA by promoting p62-LC3B-mediated autophagic degradation of TRAPβ.

### UL42 collaborates with UL31 or UL82 antagonize HCMV-induced antiviral immune responses

Previous studies have demonstrated that UL31 and UL82 respectively targeted cGAS and MITA to inhibit HCMV-induced the transcription of downstream antiviral genes. Above results suggest that UL42 targets both cGAS and MITA to suppress HCMV-triggered host antiviral immune responses and promote HCMV immune evasion. Reporter assays indicated that UL42 collaborates with UL31 or UL82 to inhibit cGAS-MITA-induced activation of the IFN-β promoter, ISRE, and NF-κB in HEK293T cells stably expressing MITA (HEK293T/MITA) (S5A Fig). Compared with UL42 or UL31, the cGAMP synthesis were reduced lower by Co-expression of UL42 and UL31 in S5B Fig. Moreover, while both UL42 and UL82 inhibited cGAMP-induced transcription of downstream genes, the inhibition efficiency by co-expression of UL42 and UL82 was higher than individual expression of UL42 or UL82 (S5C Fig). Conversely, UL42-deficiency collaborated with UL31- or UL83-deficiency in enhancing innate immune response following HCMV infection (S5D & S5E Fig). These results suggest that UL42 cooperated with UL82 and UL31 to inhibit innate immune response against HCMV.

## Discussion

The innate immune system constitutes the first line of host defense against viral infection [47]. The ability of viruses to evade and modulate host innate immune response is of central importance for successful establishment and maintenance of infection [48]. As the cGAS-MITA-TBK1 axis plays a crucial role in host defense against DNA viruses [29], the DNA viruses have evolved various mechanisms to antagonize this signaling pathway for replication and latent infection [30]. For example, HSV-1 tegument protein UL37 has been reported to deamidate cGAS which impairs the ability of cGAS to catalyze cGAMP synthesis [49]; UL41directly degrades cGAS mRNA to inhibit antiviral signaling [50]; ICP27 targets the TBK1-activated MITA/STING signalosome to inhibit antiviral response [51]. Kaposi sarcoma herpesvirus (KSHV) protein ORF52 and cytoplasmic isoforms of LANA counteract cGAS-mediated signaling [52, 53]; vIRF1 inhibits antiviral gene expression by impeding interaction of MITA with TBK1 [54].

One important feature of HCMV is to establish long-term latent infection *in vivo*. Therefore, it is understandable that HCMV may employ multiple and even redundant mechanisms to inhibit innate immune response. Previously, it has been shown that HCMV tegument protein UL83 interacts with cGAS and IFI16 in the nucleus to inhibit type I IFN induction [33, 34]; HCMV UL82 inhibits the translocation of MITA from the ER to perinuclear microsomes by disrupting the MITA-iRhom2-TRAPβ translocation complex, resulting the impairment of recruitment of TBK1 and IRF3 to the MITA complex [15]; HCMV UL31 inhibits DNA binding and enzymatic activity of cGAS, leading to decreased production of cGAMP and impairment of innate antiviral response [32]. In this study, we identified UL42 as a new HCMV protein that impairs cGAS activation and MITA trafficking, and contributing to evasion of innate immunity of HCMV. In light of these studies, it is possible that the different HCMV proteins, which are optimally expressed in the host cells at different time points or distinct intracellular locations after infection, may antagonize innate immune response in a temporal/spatial manner. In addition, cGAS activation or MITA trafficking themselves are involved in complicated regulatory mechanisms, the HCMV UL proteins may regulate distinct molecular events in these processes.

To understand the comprehensive mechanisms on how HCMV rapidly establishes persistent infection, we systematically screened for HCMV proteins that can inhibit DNA-triggered activation of ISRE [15, 32]. In this report, we investigated the role of HCMV UL42, one of the non-essential genes for HCMV viral replication [35], in antagonizing innate antiviral response. Several lines of evidence suggest that UL42 acts to antagonizing cGAS-MITA-mediated innate antiviral response. Firstly, overexpression of UL42 inhibited cGAS-induced activation of the IFN-β promoter and ISRE. Consistently, UL42 inhibited HCMV- or cytosolic dsDNA-induced transcription of downstream effector genes, whereas deficiency of UL42 increased HCMV-triggered production of type I IFNs and downstream antiviral genes. Additionally, the viral titers of HCMV-ΔUL42 were decreased in comparison with wild-type HCMV in HFFs. Although UL42 efficiently inhibits cGAS-MITA-mediated signaling, UL42-deficiency only led to a moderate increase of IRF3 activation and induction of downstream antiviral genes. The simplest explanation is that HCMV encodes multiple proteins to antagonize innate antiviral response, therefore, deficiency of one of this protein has only partial effect.

Mechanistic studies suggest that UL42 inhibits innate antiviral response by targeting both cGAS and MITA. Firstly, overexpression of UL42 inhibited HCMV-triggered induction of cGAMP and cGAMP-induced transcription of downstream effector genes. Cellular and biochemical experiments indicated that UL42 interacted with cGAS and MITA following HCMV infection. Second, *in vitro* pull-down analysis showed that UL42 inhibited the binding of cGAS to dsDNA. In mammalian cells, overexpression of UL42 inhibited the self-association of cGAS. Third, confocal microscopy revealed that UL42 impaired the trafficking of MITA, a critical process for MITA activation. Biochemical experiments indicated that UL42 impaired the trafficking of MITA by promoting p62-LC3B-mediated autophagic degradation of TRAPβ, which is a critical component in the translocon complex. Collectively, our results suggest that UL42 antagonizes innate antiviral response by inhibiting cGAS activation, as well as promoting p62-LC3B-mediated degradation of TRAPβ and therefor impairing MITA trafficking and activation. Thus, UL42 represents a new player involved in HCMV evasion of innate antiviral response.

## Materials and methods

### Reagents and antibodies

2’ 3’-cGAMP, and lipofectamine 2000 (Invitrogen); polybrene (Millipore); puromycin and RNase inhibitor (Thermo); dual-specific luciferase assay kit (Promega); SYBR (BIO-RAD); digitonin (Sigma); streptavidin agarose (Solulink); mouse antibodies against Flag, and β-actin (Sigma), and HA (Covance); rabbit monoclonal antibodies against cGAS (66546S/31659S), MITA (13647S), phosphor-MITA (85735S), phosphor-p65, and phosphor-IRF3 (4947S) (Cell Signaling Technology), phosphor-TBK1(ab109272) and TBK1(ab40676) (Abcam), IRF3 (sc-9082), phosphor-Tyrosine701-STAT1(9167S) and STAT1(sc-346) (Santa Cruz Biotechnology) were purchased from the indicated manufacturers. Antisera against UL42, UL82, and UL44 were generated by immunizing rabbits or mice with purified recombinant UL42, UL82, and UL44 proteins.

### Cells

HEK293 cells and MRC5 cells were obtained from ATCC. HFFs were provided by Dr. Min-Hua Luo (Wuhan Institute of Virology, CAS). MITA^-/-^ MLF-MITA-Flag cells were previously described [13, 52]. These cells were cultured in DMEM (Hyclone) supplemented with 10% fetal bovine serum (Gibco) and 1% penicillin–streptomycin (Thermo Fisher Scientific) at 37°C with 5% CO2. All cells were negative for mycoplasma.

### Viruses

HCMV (AD169) and HSV-1-GFP were provided by Dr. Min-Hua Luo (Wuhan Institute of Virology, CAS) and Dr. Chun-Fu Zheng (Suzhou University) respectively. HCMV-GFP was provided by Dr. Dong Yu (Washington University). HSV-1 (KOS strain) and VACV (Tian-Tan Strain) were obtained from China Center for Type Culture Collection, Wuhan, China. HCMV and HCMV-GFP stocks were prepared on HFFs and the virus titers were determined by standard TCID50 assays. HSV-1-GFP stock was prepared in Vero cells and the virus titers were determined by standard plaque assays.

### Constructs

Expression plasmids for HA-, FLAG-, MyC-, RFP- or GFP-tagged UL42, HA-, FLAG- or RFP-tagged cGAS and its truncation mutants, HA-, FLAG- or RFP-tagged MITA and its truncation mutants were constructed by standard molecular biology techniques. Expression plasmids for HA- and FLAG-tagged MDA5, TRAPβ, TBK1 and IRF3, p62, LC3B, LC3A, IKKα, IKKβ, TRIM38, TRIM32, TRIM14, iRhom2 and the IFN-β promoter reporter plasmids were previously described [13, 15, 38, 55].

### DNA oligonucleotides

The following oligonucleotides were used to stimulate cells:

ISD45:

5’-TACAGATCTACTAGTGATCTATGACTGATCTGTACATGATCTACA-3’;

VACV70: 5’-CCATCAGAAAGAGGTTTAATATTTTTGTGAGACCATGGAAGAGAGAAAGAGATAAAACTTTTTTACGACT-3’;

dsDNA90: 5’-TACAGATCTACTAGTGATCTATGACTGATCTGTACATGATCTACATACAGATCTACTAGTGATCTATGACTGATCTGTACATGATCTACA-3’;

HSV120: 5’-AGACGGTATATTTTTGCGTTATCACTGTCCCGGATTGGACACGGTCTTGTGGGATAGGCATGCCCAGAAGGCATATTGGGTTAACCCCTTTTTATTTGTGGCGGGTTTTTTGGAGGACTT-3’.

### Transfection and reporter assays

Transfection and reporter assays were performed as previously described [46]. HEK293 cells were transfected by standard calcium phosphate precipitation method. HFFs were transfected by Lipofectamine 2000. To ensure that each transfection receives the same amount of total DNA, the empty control plasmid was added to each transfection. To normalize for transfection efficiency, pRL-TK (*Renilla* luciferase) reporter plasmid (0.01 μg) was added to each transfection. Luciferase assays were performed using a Dual-Specific Luciferase Assay Kit. Firefly luciferase activities were normalized on the basis of *Renilla* luciferase activities.

### RNAi

Double-stranded oligonucleotides corresponding to the target sequences were cloned into the pSuper.Retro-RNAi plasmid (Oligoengine). The following sequences were targeted for UL42 mRNA: #1 5’-GCTGGTGGACCTCAACAACTT-3’; #2 5’-GCCAATGGATCATGCTGTTTC-3’; The following sequences were targeted for UL82: 5’-GCTGGTGGACCTCAACAACTT-3’; The following sequences were targeted for UL31: 5’-GGACAACTTTCTCACGTCT-3’; The sequence targeted by the control RNAi plasmid is: 5’-GGAAGATGTATGGAGACATGG-3’.

### shRNA-transduced stable cells

The HEK293T cells were transfected with two packaging plasmids (pGAG-Pol and pVSV-G) together with a control, UL42-, UL82-, UL31, RIG-I-, TLR9-, p62-, or LC3B-shRNA retroviral plasmid. Twenty-four hours later, cells were incubated with new medium without antibiotics for another 24 hr. The recombinant virus-containing medium was filtered and then added to HFF or HEK293 cells in the presence of polybrene (6 μg/ml). The infected cells were selected with puromycin (0.5–1.0 μg/ml) for 10 days before additional experiments.

### Coimmunoprecipitation and immunoblot analysis

HEK293 cells, or HFF cells were lysed in l ml NP-40 lysis buffer (20 mM Tris-HCl [pH 7.4], 150 mM NaCl, 1 mM EDTA, 1% Nonidet P-40, 10 μg/ml aprotinin, 10 μg/ml leupeptin, and 1 mM phenylmethyl sulfonyl fluoride). Coimmunoprecipitation and immunoblot analysis were performed as previously described [56, 57]

### GST Pull-Down assay

GST-cGAS were bound to glutathione agarose beads and incubated for 3 hrs with lysates from HEK293T cells transiently expressing HA-cGAS or UL42-HA plasmid. The beads were washed three times each with lysis buffer (20 mM Tris-HCl [pH 7.4], 150 mM NaCl, 1mM EDTA, 1% Nonidet P-40, 10 μg/ml aprotinin, 10 μg/ml leupeptin, and 1mM phenylmethyl sulfonyl fluoride), then mixed with an equal volume of 2× SDS loading buffer and boiled for 10 min. The input/elutes were resolved by SDS-PAGE and analyzed by coomassie staining and/or immunoblot analysis [52].

### *In vitro* pull-down assay

HEK293 cells transfected with the indicated plasmids were lysed in NP-40 lysis buffer. Lysates were incubated with biotinylated-HSV120 for 1 hour at 4°C, and then incubated with streptavidin beads for another 2 hours at 4°C. The beads were washed three times with lysis buffer and analyzed by immunoblotting with the indicated antibodies.

### Digitonin permeabilization

Cells were mock-transfected or transfected with HSV120 (3 μg/ml) for 4 hours. Cell extracts were then prepared and heated at 95°C for 5 min to denature most proteins, which were removed by centrifugation. The supernatants containing cGAMP were delivered to MLFs pretreated with digitonin permeabilization solution (50 mM HEPES pH 7.0, 100 mM KCl, 3 mM MgCl_2_, 0.1 mM DTT, 85 mM Sucrose, 0.2% BSA, 1 mM ATP, 0.1 mM GTP and 10μg/ml digitonin) at 37°C for 30 min. Three hours later, the cells were collected for a qPCR analysis.

### CRISPR/Cas9-mediated genome editing of HCMV

The experiments were performed as previously described [58, 59]. Briefly, potential guide RNAs (gRNAs) targeting UL42 gene were analyzed using the CRISPR Design tool. The UL42 gRNA target sequence used in this study is 5’- CGTCGTCGGGCACAGACCCA-3’. Double-stranded oligos were cloned into the lentiCRISPRv1 vector and cotransfected with packaging plasmids into HEK293T cells. Lentiviral particles were collected and used to transduce HFFs. The HFF-gRNA cells were infected with serial dilution of HCMV in 96 well plates. Twenty days later, the viruses were collected and diluted to infect HFFs for 20 days. A single plaque was pick up to infect HFFs to produce homogenous HCMV-ΔUL42 strain, which was verified by immunoblotting analysis.

### qPCR

Total RNA was isolated for qPCR analysis to measure mRNA levels of the indicated genes. Data shown are the relative abundance of the indicated mRNA normalized to that of GAPDH. Primer sequences for *IFNB1*, *ISG56*, *ISG54*, *CXCL10*, *IL6*, *Uls*, and *GAPDH* were previously described [32, 38, 60].

### Fluorescent confocal microscopy

The cells were incubated with the ER-Tracker Green or Mito-Tracker Red (Invitrogen) following protocols recommended by the manufacturer. The cells were then fixed with 4% paraformaldehyde for 10 minutes and observed with an Olympus confocal microscope under a 60× oil objective.

## Supporting information

S1 Fig HCMV AD169 strain does not infect endothelial and epithelial cells, related to Fig 1. (A) The indicated cells (4x10^5^) were infected with HCMV (MOI = 1) for the indicated times before qPCR analysis.
(B) The indicated cells (4x10^5^) were infected with HCMV (MOI = 1) for the indicated times before immunoblotting analysis with the indicated antibodies.
(TIF)

S2 Fig UV treatment induces HCMV inactivation, related to Fig 2. HFF cells (4x10^5^) were infected with wild-type or UV-inactivated HCMV before qPCR analysis.
(TIF)

S3 Fig RIG-I and TLR9 are not involved in innate immune responses to HCMV, related to Fig 3. (A) Effects of MITA-deficiency on HCMV-induced transcription of downstream antiviral genes. MITA-deficient (KO) HFF cells were generated by the CRISPR-Cas9 method. MITA-KO and control HFF cells (4x10^5^) were infected with HCMV for the indicated times before qPCR analysis.
(B) Effects of RIG-I knockdown on HCMV-induced transcription of *IFNB1*. RIG-I-knockdown and control HFF cells (4x10^5^) were infected with HCMV for the indicated times before qPCR analysis.
(C) Effects of TLR9 knockdown on HCMV-induced transcription of *IFNB1*. TLR9-knockdown and control HFF cells(4x10^5^) were infected with HCMV for the indicated times before qPCR analysis.
Graphs show mean ± SD, n = 3. *p<0.05, **p<0.01 (unpaired t test).
(TIF)

S4 Fig UL42 does not affect polyubiquitination of cGAS and MITA, related to Figs 5 and 6. HEK293 cells (1x10^6^) were transfected with Flag-cGAS or Flag-MITA (2 μg each), HA-Ub or its mutants (1 μg each), and a control or UL42 expression plasmid (0.5 μg) for 20 hr, followed by co-immunoprecipitation and immunoblotting analysis with the indicated antibodies.
(TIF)

S5 Fig UL42 collaborates with UL82 and UL31 to antagonize HCMV-induced antiviral immune response. (A) UL42 collaborates with UL82 and UL31 to inhibit cGAS-MITA-mediated activation of the IFNβ promoter, ISRE and NF-κB. HEK293T-MITA cells were transfected with the IFNβ promoter (0.05 μg), ISRE (0.03 μg) or NF-κB (0.005 μg) reporter plasmid, and expression plasmids for cGAS (0,01 μg) and UL42, UL82 or UL31 (0.05 μg each) for 20 hr before luciferase assays.
(B) Effects of UL42 and UL31 on cGAMP synthesis induced by HCMV. HFF-Vec, HFF-UL42, HFF-UL31, or HFF- UL42/UL31 cells (1x10^7^) were uninfected or infected with HCMV (MOI = 3) for 5 hr, and then cell extracts containing cGAMP were delivered to digitonin-permeabilized HFFs for 4 hr before qPCR analysis.
(C) Effects of UL42 and UL82 on cGAMP-induced transcription of antiviral genes in HFFs. Control, UL42, UL82 or UL42/82-tranduced HFFs (4x10^5^) were transfected with cGAMP (0.1 μg) for 4 hr before qPCR analysis.
(D-E) Effects of knockdown of UL42, UL31, or UL82 on HCMV-induced transcription of downstream antiviral genes. UL42, UL31, UL82, UL42/31 or UL42/82 shRNA stable HFFs (4x10^5^) were infected with HCMV (MOI = 1) for the indicated times before qPCR analysis.
Graphs show mean ± SD, n = 3. *p<0.05, **p<0.01 (unpaired t test).
(TIF)
