# Supplementary figures and images for: Human cytomegalovirus protein UL42 antagonizes cGAS/MITA-mediated innate antiviral response
Source: PLoS Pathog. 2019 May 20;15(5):e1007691. doi: 10.1371/journal.ppat.1007691 (PMC6527189; doi:10.1371/journal.ppat.1007691)

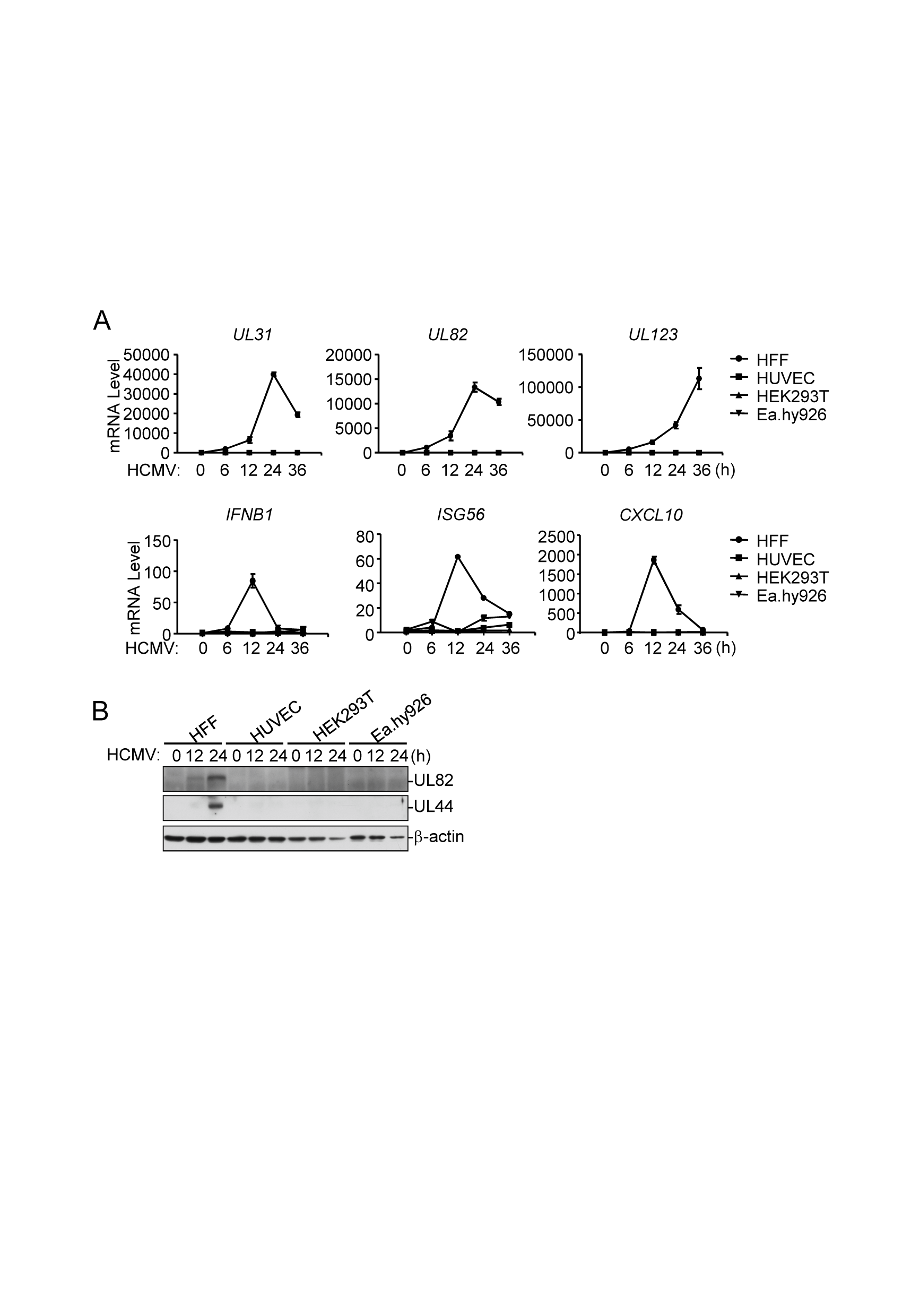

Supplement: S1 Fig — (A) The indicated cells (4x10^5^) were infected with HCMV (MOI = 1) for the indicated times before qPCR analysis. (B) The indicated cells (4x10^5^) were infected with HCMV (MOI = 1) for the indicated times before immunoblotting analysis with the indicated antibodies. (TIF) [file ppat.1007691.s001.tif]

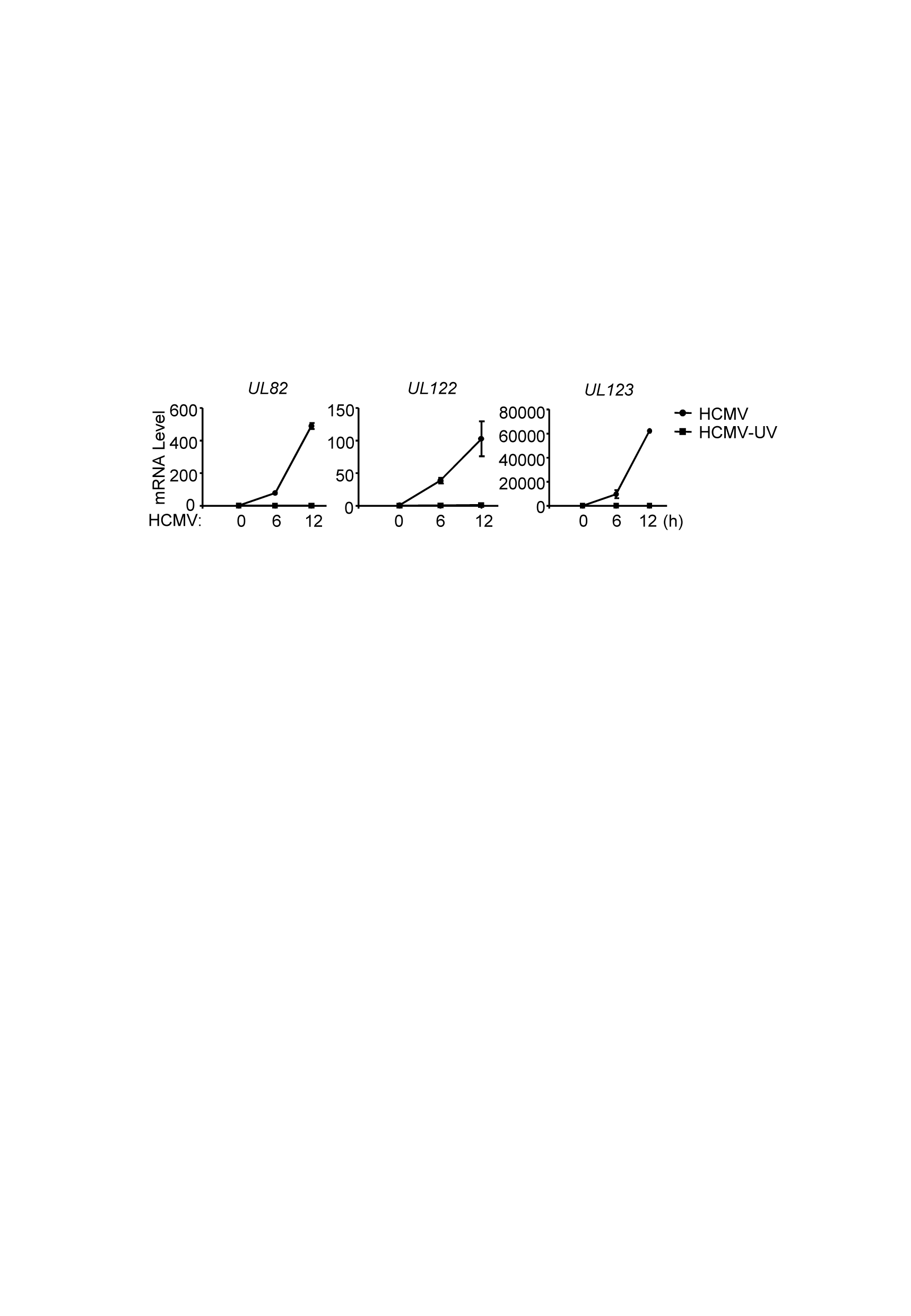

Supplement: S2 Fig — HFF cells (4x10^5^) were infected with wild-type or UV-inactivated HCMV before qPCR analysis. (TIF) [file ppat.1007691.s002.tif]

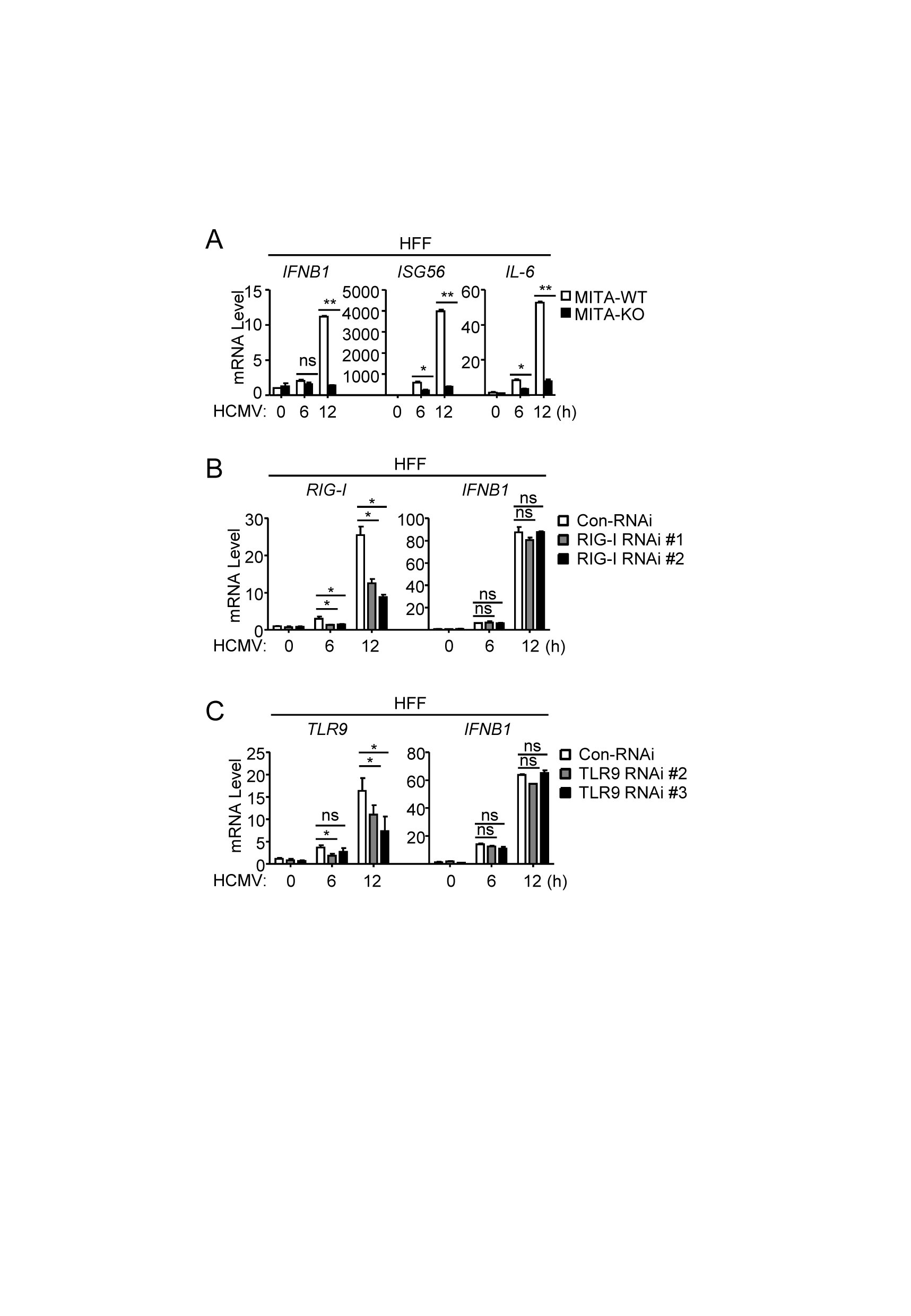

Supplement: S3 Fig — (A) Effects of MITA-deficiency on HCMV-induced transcription of downstream antiviral genes. MITA-deficient (KO) HFF cells were generated by the CRISPR-Cas9 method. MITA-KO and control HFF cells (4x10^5^) were infected with HCMV for the indicated times before qPCR analysis. (B) Effects of RIG-I knockdown on HCMV-induced transcription of IFNB1. RIG-I-knockdown and control HFF cells (4x10^5^) were infected with HCMV for the indicated times before qPCR analysis. (C) Effects of TLR9 knockdown on HCMV-induced transcription of IFNB1. TLR9-knockdown and control HFF cells(4x10^5^) were infected with HCMV for the indicated times before qPCR analysis. Graphs show mean ± SD, n = 3. *p<0.05, **p<0.01 (unpaired t test). (TIF) [file ppat.1007691.s003.tif]

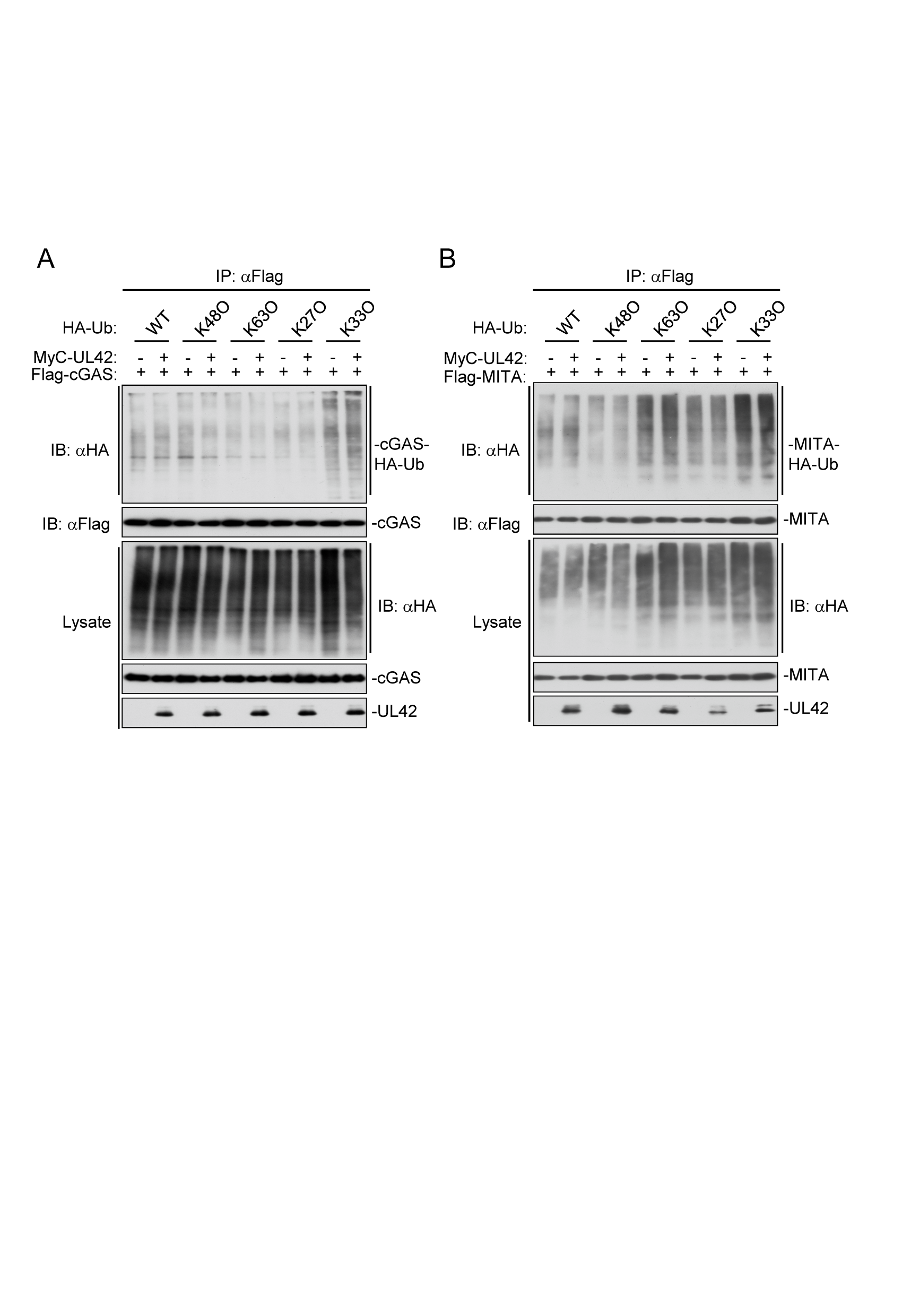

Supplement: S4 Fig — HEK293 cells (1x10^6^) were transfected with Flag-cGAS or Flag-MITA (2 μg each), HA-Ub or its mutants (1 μg each), and a control or UL42 expression plasmid (0.5 μg) for 20 hr, followed by co-immunoprecipitation and immunoblotting analysis with the indicated antibodies. (TIF) [file ppat.1007691.s004.tif]

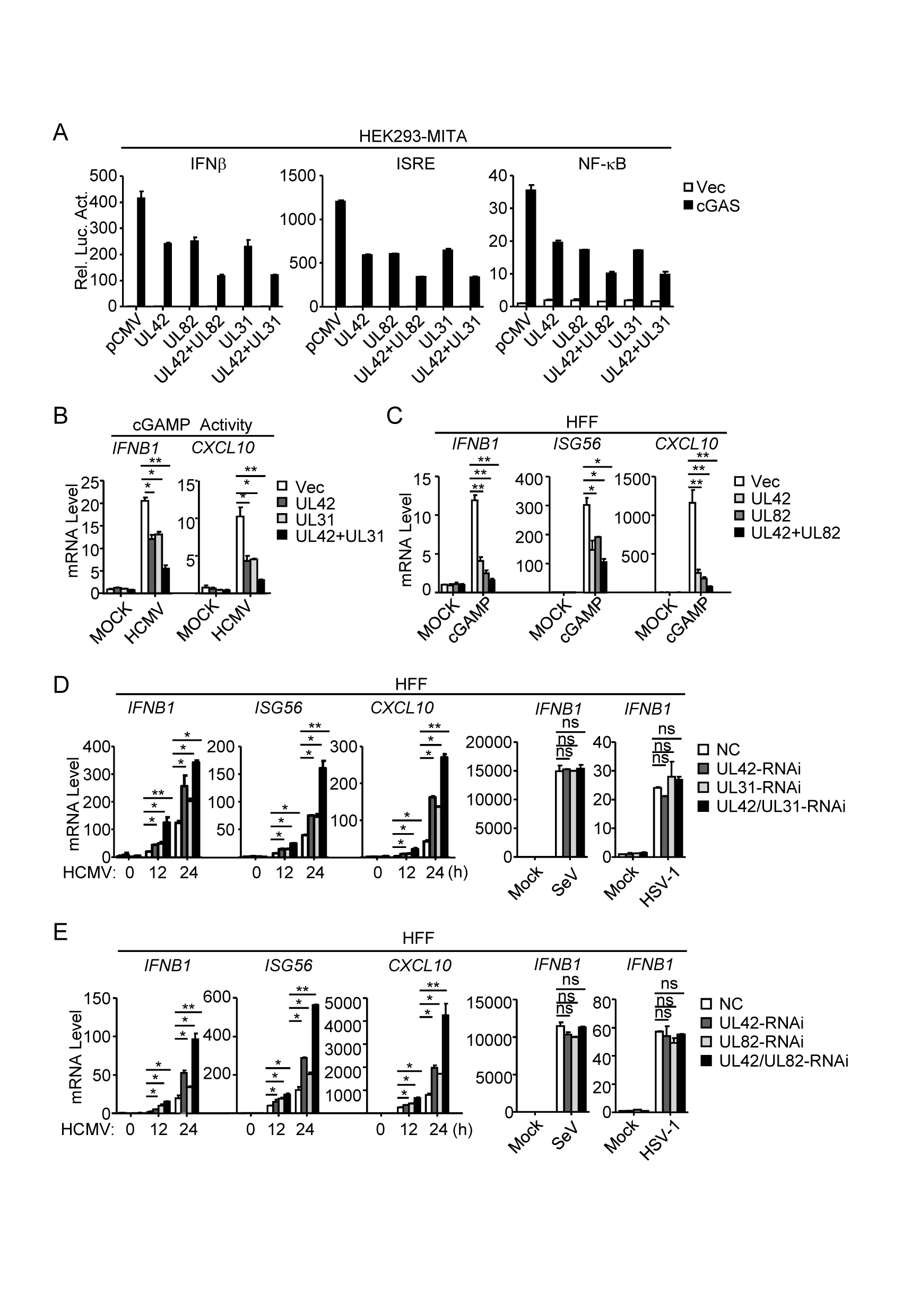

Supplement: S5 Fig — (A) UL42 collaborates with UL82 and UL31 to inhibit cGAS-MITA-mediated activation of the IFNβ promoter, ISRE and NF-κB. HEK293T-MITA cells were transfected with the IFNβ promoter (0.05 μg), ISRE (0.03 μg) or NF-κB (0.005 μg) reporter plasmid, and expression plasmids for cGAS (0,01 μg) and UL42, UL82 or UL31 (0.05 μg each) for 20 hr before luciferase assays. (B) Effects of UL42 and UL31 on cGAMP synthesis induced by HCMV. HFF-Vec, HFF-UL42, HFF-UL31, or HFF- UL42/UL31 cells (1x10^7^) were uninfected or infected with HCMV (MOI = 3) for 5 hr, and then cell extracts containing cGAMP were delivered to digitonin-permeabilized HFFs for 4 hr before qPCR analysis. (C) Effects of UL42 and UL82 on cGAMP-induced transcription of antiviral genes in HFFs. Control, UL42, UL82 or UL42/82-tranduced HFFs (4x10^5^) were transfected with cGAMP (0.1 μg) for 4 hr before qPCR analysis. (D-E) Effects of knockdown of UL42, UL31, or UL82 on HCMV-induced transcription of downstream antiviral genes. UL42, UL31, UL82, UL42/31 or UL42/82 shRNA stable HFFs (4x10^5^) were infected with HCMV (MOI = 1) for the indicated times before qPCR analysis. Graphs show mean ± SD, n = 3. *p<0.05, **p<0.01 (unpaired t test). (TIF) [file ppat.1007691.s005.tif]
